# ﻿A new species of the ant *Platythyreaclypeata* species group (Hymenoptera, Formicidae, Ponerinae) from continental Asia

**DOI:** 10.3897/zookeys.1115.86477

**Published:** 2022-08-01

**Authors:** Weeyawat Jaitrong, Zhenghui Xu, Salinee Khachonpisitsak

**Affiliations:** 1 Office of Natural Science Research, National Science Museum, 39 Moo 3, Khlong 5, Khlong Luang, Pathum Thani, 12120, Thailand National Science Museum Pathum Thani Thailand; 2 Biology Divisions, Faculty of Science and Technology, Rajamangala University of Technology Thanyaburi, Pathum Thani, 12120 Thailand Rajamangala University of Technology Thanyaburi Pathum Thani Thailand; 3 Key Laboratory of Forest Disaster Warning and Control in Yunnan Province, College of Biodiversity Conservation, Southwest Forestry University, Kunming, Yunnan Province 650224, China Southwest Forestry University Kunming China; 4 Department of Biology, Faculty of Science, Burapha University, 169 Long-Hard Bangsaen Road, Sanesuk, Mueang, Chon Buri, 20131 Thailand Burapha University Chon Buri Thailand

**Keywords:** ant, distribution, new species, species group, Thailand

## Abstract

The *Platythyreaclypeata* species group is reviewed and three species, including one new species, *P.homasawini***sp. nov.**, are recognized. This species group is distinguished from the *P.parallela* species group by the reddish-brown body, the elliptical shape of the propodeal spiracle, the elongate antennal scape, and the distinctly narrowed posteriad space between frontal carinae. *Platythyreahomasawini***sp. nov.**, from Thailand and China, is described based on the worker caste. The type series of the new species was collected on the forest floor from dead wood in an advanced stage of decomposition. A key to the Oriental species of the genus *Platythyrea* based on the worker caste is provided.

## ﻿Introduction

The ant genus *Platythyrea* Roger, 1863 (Formicidae, Ponerinae) is a rarely collected group with recent reviews listing 39 species (Schmidt and Shattuck 2014; Antweb 2022). *Platythyrea* has small colonies usually with a few hundred workers or fewer (Villet et al. 1990; Villet 1991; Ito 1994, 2016; Djiéto-Lordon et al. 2001; Hartmann et al. 2005; Molet and Peeters 2006; Yéo et al. 2006). The genus is mainly and widely distributed within tropical and subtropical regions, with peak diversity in the Ethiopian region (17 spp.), followed by the Neotropical (9 spp.), Oriental (9 spp.), and Australian regions (6 spp.) (Schmidt and Shattuck 2014; Bolton 2022). At present, seven species have been recorded in Southeast Asia (*Platythyreabidentata* Brown, 1975; *P.clypeata* Forel, 1911; *P.inermis* Forel, 1910; *P.parallela* (F. Smith, 1859); *P.quadridenta* Donisthorpe, 1941; *P.tricuspidata* Emery, 1900 and *P.janyai* Phengsi, Jaitrong, Ruangsittichai & Khachonpisitsak, 2018). Among them, five species are reported from Thailand, and they belong to two species groups (sensu Brown 1975): the *P.clypeata* group (*P.clypeata* and *P.janyai*) and *P.parallela* group (*P.parallela*, *P.quadridenta*, and *P.tricuspidata*) (Phengsi et al. 2018; Khachonpisitsak et al. 2020). Our recent examination of *Platythyrea* specimens from Thailand, Laos, Malaysia, and China has revealed the presence of a new species assigned to the *P.clypeata* group. The distribution data and diagnostic features for three species in this group are discussed. A key to the Oriental species of the genus *Platythyrea* based on the worker caste is updated from Phengsi et al. (2018).

## ﻿Materials and methods

This study is mainly based on the material deposited in the Natural History Museum of the National Science Museum, Thailand (THNHM). The specimens were compared with high-resolution images of holotypes and paratypes of closely related species (*Platythyreagracillima* Wheeler, 1922; *P.clypeata*; and *P.prizo* Kugler, 1977) available on Antweb (2022) and Antwiki (2022). The holotype and paratypes of *P.janyai* were also examined. The holotype and paratypes of *P.homasawini* sp. nov. are deposited in THNHM.

Most morphological observations were made with a ZEISS Discovery V12 stereoscope. Multi-focused montage images were produced using NIS-Elements-D from a series of source images taken by a Nikon Digital Sight-Ri1 camera attached to a Nikon AZ100M stereoscope. Specimens were measured for the following parts using a micrometer on a ZEISS Discovery V12 stereoscope. All measurements are given in millimeters and recorded to the second decimal place.

Standard measurements and indices used in the paper are as defined in Bolton (1975):

**CI** Cephalic index = HW/HL ×100.

**DPW** Dorsal petiole width: maximum width of petiole in dorsal view.

**EI** Eye index = EL/HW ×100.

**EL** Eye length: the maximum length of the eye.

**HL** Head length: straight-line length of head in perfect full-face view, measured from the mid-point of the anterior clypeal margin to the midpoint of the posterior margin. In species where one or both of these margins are concave, the measurement is taken from the mid-point of a transverse line that spans the apices of the projecting portions.

**HW** Head width: maximum width of head in full-face view, excluding the eyes.

**ML** Mesosoma length: the diagonal length of the mesosoma in lateral from the anterior margin of the pronotum to the posteroventral angle of the metapleuron, excluding the neck.

**PH** Petiole height: height of petiole measured in lateral view from the apex of the ventral (subpetiolar) process vertically to a line intersecting the dorsal most point of the node.

**PL** Petiole length: length of petiole measured in lateral view from the anterior articulation to the posterior articulation of petiole.

**PW** Pronotal width: maximum width of pronotum measured in dorsal view.

**SL** Scape length: straight-line length of the antennal scape, excluding the basal constriction or neck.

**SI** Scape index = SL/HW ×100.

**TL** Total length: total outstretched length of the individual, from the mandibular apex to the gastral apex.

Abbreviations of the type depositories are as follows:

**BMNH** The Natural History Museum, London, U.K.

**MHNG**Muséum d’Histoire Naturelle, Geneva, Switzerland.

**THNHM** Natural History Museum of the National Science Museum, Thailand.

Scanning electron microscope images of *Platythyrea* species were made at the Microscopic Center, Faculty of Science, Burapha University with a LEO 1450 VP scanning electron microscope on gold coated specimens.

## ﻿Taxonomy

### ﻿*Platythyreaclypeata* species group

**Figure 1.**
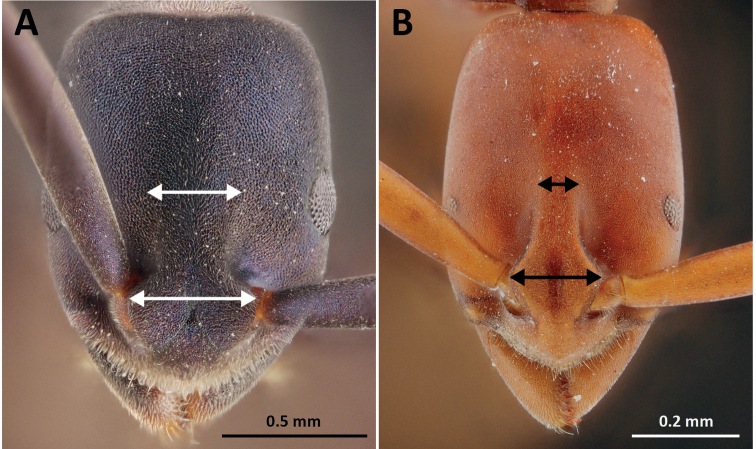
Frontal view focusing on the space between frontal carinae. **A** frontal carinae very widely spaced **B** frontal carinae relatively narrowly separated.

The species group can be characterized by the following characteristics: 1) body reddish brown; 2) space between frontal carinae distinctly narrowed posteriad (compare Fig. 1B with Fig. 1A); 3) masticatory margin of mandible triangular with a large apical tooth, followed by 9 or 10 smaller teeth, with large and smaller teeth alternating; 4) propodeal spiracle opening elliptical; and 5) posterior margin of petiole convex without spines in dorsal view. Three species are recognized in continental Asia.

#### ﻿ Platythyrea clypeata

Taxon classificationAnimaliaHymenopteraFormicidae

Forel, 1911

ED5F2E00-8A46-56BF-9E4F-D09DCF33DF73

Figs 2 , 5A1–A3

Platythyrea clypeata Forel, 1911: 378; Brown 1975: 50; Bolton 1995: 336; Schmidt and Shattuck 2014: 51; Khachonpisitsak et al. 2020: 153. Senior synonym of P.thwaitesi: Brown 1975: 8.
Platythyrea thwaitesi Donisthorpe, 1931: 496. Junior synonym of P.clypeata: Brown 1975: 8.

##### Type.

***Holotype*** of *P.clypeata*: alate queen from “Pays des Moïs”, Cochinchinne française (Indochina), deposited in MHNG (AntWeb image examined, CASENT0907112). Holotype of *P.thwaitesi*: alate queen from Sri Lanka, deposited in BMNH (AntWeb image examined, CASENT0900568).

##### Non-type material examined.

Laos • 10 workers (THNHM-I-24983) and 1 dealate queen (THNHM-I-24982), Laos, Vientiane, Pak Ngum District, Ban Phang Dang, ca 300 m alt., 14.VI.2010, W. Jaitrong leg., Colony no. WJT-LAO-143 (THNHM-I-24981) • 1 worker (THNHM-I-24984), Laos, Vientiane, Pak Ngum District, Ban Phang Dang, 14.VI.2010, Sk. Yamane leg., Colony no. LA10-SKY-128 • 1 worker (THNHM-I-24981) Laos, Vientiane, Pak Ngum District, Ban Phang Dang, ca 300 m alt., 12.VI.2010, W. Jaitrong leg. – Thailand • 9 workers (THNHM-I-02423 to THNHM-I-02431), eastern Thailand, Chachoengsao Province, Tha Takiab District, Khao Ang Reu Nai Wildlife Sanctuary, 27.IX.2002, W. Jaitrong leg., Colony no. WJT270902-01 • 3 workers (THNHM-I-02432 to THNHM-I-02434), same locality, date and collector, Colony no. WJT270902-1 • 6 workers (THNHM-I-02435 to THNHM-I-02440), eastern Thailand, Sa Kaeo Province, Khao Ang Reu Nei Wildlife Sanctuary, 26.VI.2003, W. Jaitrong leg., Colony no. WJT03-TH-228; 22 workers (THNHM-I-02441 to THNHM-I-02452) and 1 male (THNHM-I-02453), eastern Thailand, Chanthaburi Province, Soi Dao District, 14.V.2008, W. Jaitrong leg., Colony no. WJT08-E065 • 14 workers (THNHM-I-24980, THNHM) and 1 dealate queen, central Thailand, Nakhon Nayok Prov., Muang Dist., Nang Rong Temple, in rotting wood, 29.VIII.2018, W. Jaitrong leg., Colony no. WJT290819-09.

**Figure 2.**
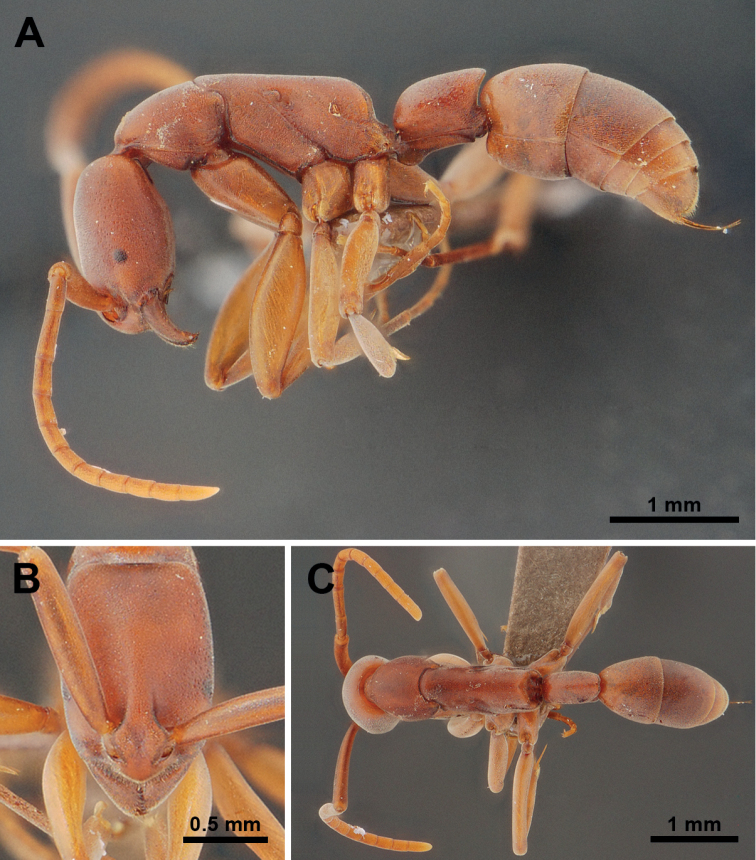
*Platythyreaclypeata* (non-type worker, WJT290819-09). **A** body in profile view **B** head in full-face view **C** body in dorsal view.

##### Measurements and indices

**(*n* = 10).**TL 5.74–6.20, HL 1.29–1.39, HW 0.86–0.89, SL 1.12–1.18, EL 0.10, PW 0.76–0.83, ML 1.85–2.05, PL 0.66–0.73, PH 0.46–0.53, DPW 0.40–0.43, CI 61–69, EI 11, SI 125–138.

##### Description of workers.

***Head*** in full-face view rectangular, clearly longer than broad, with sides weakly convex or almost parallel, posterior corners narrowly rounded, posterior margin feebly concave. Antennae relatively short, scapes extending beyond posterior corners of head by about 1/5 of their length. Clypeus roughly triangular, median portion distinctly convex, anterior margin bluntly angled. Mandibles triangular, masticatory margin with a large apical tooth, followed by about ten smaller teeth, large and small teeth alternating, basal margin without denticle. Eye very small and flat, located laterally at anterior to mid-length of head, with five ommatidia along longest axis. Frontal lobes close to each other and rounded. Space between frontal carinae distinctly narrowed posteriad.

***Mesosoma*** elongate, in profile pronotum weakly convex, promesonotal suture distinct, dorsal outline of mesonotum and propodeum straight, metanotal groove absent, propodeal junction broadly rounded, declivity of propodeum obviously concave, propodeal spiracle opening elliptical. Legs long.

***Petiole*** cylindrical in profile view and sessile; in profile view clearly longer than high, dorsal outline almost straight, anterodorsal corner broadly rounded, posterodorsal corner forming an acute angle, posterior margin weakly concave, ventral margin weakly concave with narrowly rounded anteroventral corner; in dorsal view the node roughly rectangular, longer than broad, slightly narrower posteriorly, posterior margin convex and with shallow median concavity.

***Sculpture*.** Dorsum of head finely punctate, lateral face of head above eyes punctate, with dense foveae. Dorsum of mesosoma finely punctate similar to dorsum of head, lateral faces of pronotum, metapleuron and propodeum punctate, with sparse shallow foveae. Petiole finely micropunctate. Gastral tergites I and II finely reticulate. Antennal scapes finely micropunctate.

***Pilosity*.** Pubescence very short and fine all over the body surface; standing hairs present on tip of gaster.

***Coloration*.** Body color reddish brown to darkish brown; antennae, legs, and tip of gaster yellowish brown to reddish brown. Eyes grey. Pilosity white.

##### Description of dealate queen.

See Phengsi et al. (2018).

##### Habitat.

*Platythyreaclypeata* occurs in the lowlands (200–300 m alt.) and inhabits primary dry evergreen forest and disturbed forests. All colonies of this species were collected from dead wood on the forest floor in an advanced stage of decomposition.

##### Distribution.

Sri Lanka, Vietnam, Laos (Vientiane), and Thailand (Chachoengsao, Sa Kaeo, Chanthaburi, and Nakhon Nayok provinces) (Fig. 6).

##### Comparative notes.

*Platythyreaclypeata* is similar to *P.gracillima*, *P.janyai*, and *P.prizo*. However, *P.clypeata* can be easily separated from *P.gracillima* by the following characteristics (characters of *P.gracillima* in parentheses unless otherwise stated): 1) body size smaller (TL = 5.74–6.20 in *P.clypeata*; TL = 9 mm in *P.gracillima*); 2) eye smaller and flat (0.1 mm long in *P.clypeata*; 0.3 mm long and convex in *P.gracillima*); 3) clypeus narrow and rather convex (clypeus broad and rather flat); 4) declivity of propodeum concave (propodeal declivity flat); 5) seen from back propodeal declivity rounded above (propodeal declivity concave above); 6) petiole laterally swollen (petiole laterally compressed); 7) head finely punctate (head rather smooth).

*Platythyreaclypeata* can be distinguished from *P.prizo* by the following characteristics (characters of *P.prizo* in parentheses unless otherwise stated): 1) head shorter (CI = 61–69 in *P.clypeata*; CI = 70–72 in *P.prizo*); 2) eye flat and small (0.1 long mm in *P.clypeata*; eye very large, 0.31 long mm *P.prizo*); 3) eye without erect pubescence (eye covered with extremely fine short erect pubescence); 4) propodeum junction obtusely angulated (propodeum junction armed with a pair of short teeth or tubercles); 5) in profile, posterodorsal corner of petiole forming an acute angle (roundly convex).

Lastly, this species is separated from *P.janyai* (characters of *P.janyai* in parentheses unless otherwise stated) by 1) head relatively longer (CI 61–69 in *P.clypeata*; CI 72–74 in *P.janyai*); 2) eye clearly smaller (EL 0.10 mm long with 5 ommatidia on longest axis in *P.clypeata*; EL 0.20 mm long with 11 ommatidia on longest axis in *P.janyai*); 3) eye flat (eye convex); 4) head finely punctate with dense shallow foveae (dorsum and lateral face of head finely micropunctate without foveae); 5) in profile petiole slightly longer than high and in dorsal view its node slightly narrower posteriorly (clearly longer than high and in dorsal view node anteriorly as broad as posteriorly); 6) ventral outline of petiole feebly concave (weakly convex) (see Fig. 5 for comparison).

#### ﻿ Platythyrea homasawini sp. nov.

Taxon classificationAnimaliaHymenopteraFormicidae

B6867BBB-982B-524B-A5CB-48BEE37EE4BA

https://zoobank.org/B91E483E-24FB-454F-9F14-8EAC635F5631

Figs 3 , 5B1–B3

Platythyrea clypeata : Xu and Zeng 2000: 214, figs 1–3.

##### Type.

***Holotype*** worker (THNHM-I-26225), northern Thailand, Chiang Mai Prov., Doi Saket Dist., Ban Mae Pong, 24.XI.2021, K. Homasawin leg., Colony No. WJT241121-01. ***Paratypes***: 29 workers (THNHM-I-26226 to THNHM-I-26250, THNHM-I-24977 to THNHM-I-24979, and THNHM-I-26445), same data as holotype.

##### Non-type material examined.

China • 1 worker, Yunnan Prov., Menghai County, Meng’a Town, Papo Village, 1280 m, No. A97-2318, collected from secondary monsoon evergreen broadleaf forest, 10.IX.1997, Zhenghui Xu leg. – Thailand • 1 worker (THNHM-I-02463), northern Thailand, Chiang Mai Prov., Muang Dist., restored forest, 8.V.2002, S. Sonthichai leg. • 1 worker (THNHM-I-02454), western Thailand, Kanchanaburi Prov., Thong Pha Phum N.P., Natural Forest, 8.III.2005, W. Sakchooeong leg.

##### Measurements and indices.

***Holotype*.**TL 7.72, HL 1.60, HW 1.08, SL 1.72, EL 0.20, PW 0.96, ML 2.84, PL 1.08, PH 0.68, DPW 0.44, CI 68, EI 19, SI 159. ***Paratypes*** (*n* = 10). TL 7.72–7.80, HL 1.56–1.64, HW 1.08–1.12, SL 1.64–1.68, EL 0.16–0.20, PW 0.96–0.99, ML 2.80–2.84, PL 1.08–1.12, PH 0.60–0.64, DPW 0.40–0.44, CI 68–69, EI 15–19, SI 150–159.

##### Description of workers

**(*holotype* and *paratypes*). *Head*** in full-face view subrectangular, clearly longer than broad, weakly widening anteriorly, sides weakly convex, posterior margin almost straight, posterior corners narrowly rounded. Antennae relatively long, scapes extending beyond posterior corners of head by about 1/4 of their length. Clypeus roughly triangular, roundly convex medially, anterior margin bluntly angled. Mandibles triangular, masticatory margin with a large apical tooth, followed by eight smaller teeth, large and small teeth alternating, basal margin without denticle. Eyes flat, located at anterior 1/3 of head length, moderately large, each with 7 or 8 ommatidia along its longest axis. Frontal lobes close to each other and rounded. Space between frontal carinae distinctly narrowed posteriad.

**Figure 3.**
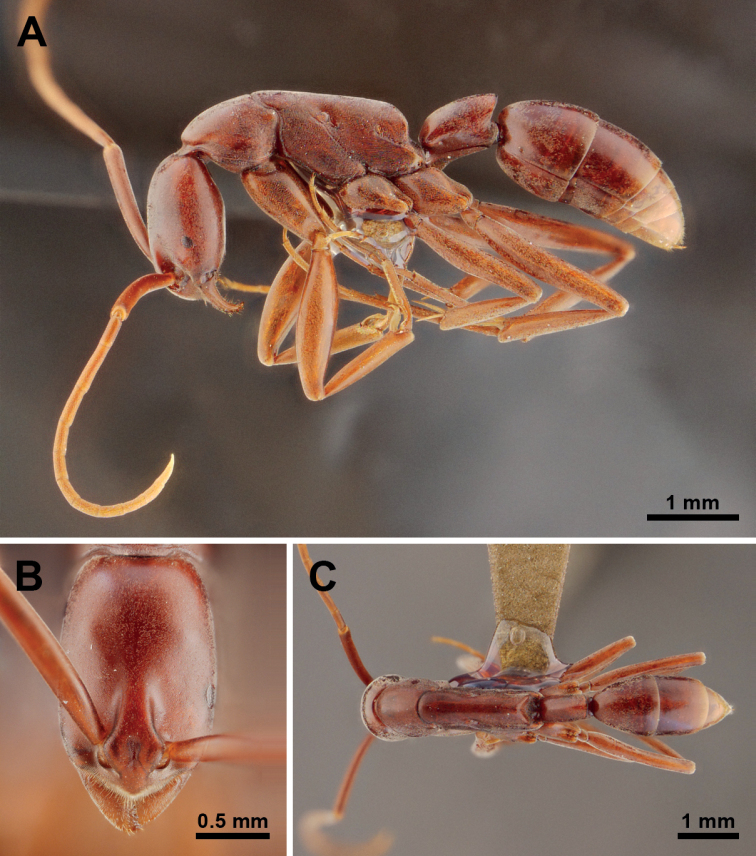
*Platythyreahomasawini* sp. nov. (holotype worker, THNHM-I-02463). **A** body in profile view **B** head in full-face view **C** body in dorsal view.

***Mesosoma*** elongate, in profile view pronotum weakly convex, promesonotal suture distinct, dorsal outline of mesonotum and propodeum straight, metanotal groove absent, propodeal junction broadly rounded, declivity of propodeum obviously concave; propodeal spiracle opening elliptical. Legs very long.

***Petiole*** cylindrical and sessile, in profile view clearly longer than high, dorsal outline weakly convex, anterodorsal corner broadly rounded, posterodorsal corner extending into an acute angle, posterior margin oblique and straight, ventral margin almost straight with narrowly prominent anteroventral corner; in dorsal view the node of petiole rectangular, longer than broad, sides straight and parallel, posterior margin weakly convex.

***Sculpture*.** Head entirely finely micropunctate. Dorsum of mesosoma finely micropunctate, mesopleuron, metapleuron and sides of propodeum punctate. Petiole finely micropunctate. Gaster superficially shagreened.

***Pilosity*.** Whole body surface and appendages covered with very short thin pubescence. Standing hairs absent.

***Coloration*.** Body color reddish brown; antennae, legs, and tip of gaster yellowish brown. Eyes grey. Pubescence white.

##### Distribution.

China (Yunnan Province) and Thailand (Chiang Mai and Kanchanaburi provinces) (Fig. 6).

##### Etymology.

The specific name is dedicated to Mr Kaisihanat Homasawin, who donated the type series.

##### Comparative notes.

The new species is similar to *P.janyai* but it can be separated from *P.janyai* by the following characteristics (characters of *P.janyai* in parentheses unless otherwise stated): 1) head weakly widening anteriorly (head not widening anteriorly); 2) dorsal outline of petiole weakly convex (dorsal outline of petiole almost straight); 3) posterior margin of petiolar node without a concavity in the middle (posterior margin of petiolar node with a concavity in the middle): 4) body surface with thin pubescence (body surface with thick pubescence); 5) head longer (CI = 66 in *P.homasawini*, CI = 72 in *P.janyai*); 6) antennal scape relatively long, 1/3 of its length extending beyond posterolateral corner of head (clearly extending beyond posterolateral corner of head); 7) eye smaller and flat (EL = 0.17 mm in *P.homasawini*; EL = 0.20 mm and slightly convex in *P.janyai*); 8) seen from back propodeal declivity rounded above (propodeal declivity tapering above); 9) dorsal outline of petiole weakly convex (almost straight).

*Platythyreahomasawini* can be easily separated from *P.clypeata* by the following characteristics (characters of *P.clypeata* in parentheses unless otherwise stated): 1) head in full-face view, posterior margin weakly concave (posterior margin almost straight); 2) antennal scape relatively long, 1/3 of its length extending beyond posterolateral corner of head (antennal scape short, 1/4 of its length extending beyond posterolateral corner of head); 3) clypeus broad (clypeus narrow); 4) eye larger (EI = 15, with 7 ommatidia in *P.homasawini*; EI = 11, with 5 ommatidia in *P.clypeata*); 5) mesosoma relative longer (WL = 2.64 in *P.homasawini*; WL=1.85–2.05 in *P.clypeata*); 6) mesopleuron not demarcated from mesonotum (mesopleuron clearly demarcated from mesonotum by shallow furrow); 7) in profile view, propodeal junction roundly convex (propodeal junction obtusely angulate); 8) in dorsal view, posterior margin of petiole clearly convex (posterior margin of petiole convex with shallow median concavity); 9) lateral face of head entirely micropunctate (lateral face of head areas behind, above and below eye punctate, with dense foveae); 10) gaster superficially shagreened (finely reticulate).

*Platythyreahomasawini* can be distinguished from *P.gracillima* by the following characteristics (characters of *P.gracillima* in parentheses unless otherwise stated): 1) clypeus roundly convex (rather flat in *P.gracillima*); 2) petiole laterally convex; 3) seen from above longer than high (petiole laterally compressed); 4) seen from above a little more than twice as long as broad); 5) mandible finely micropunctate (mandible finely and densely punctate); 6) head entirely finely micropunctate (rather smooth in *P.gracillima*).

#### ﻿ Platythyrea janyai

Taxon classificationAnimaliaHymenopteraFormicidae

Phengsi, Jaitrong, Ruangsittichai & Khachonpisitsak, 2018

80F1B0B2-8F43-5645-B08A-D5F34E0D516D

Figs 4 , 5C1–C3

Platythyrea janyai Phengsi et al. 2018: 89, figs 1, 5B1–B3; Khachonpisitsak et al. 2020: 154.

##### Types.

***Holotype*** (THNHM-I-02392) and three ***paratypes*** (THNHM-I-02393 to THNHM-I-02395) workers, southern Thailand, Phatthalung Province, Si Banphot District, Riang Thong Waterfall, Khao Pu Khao Ya National Park, 28.IX.2007, W. Jaitrong leg., Colony no. WJT07-TH-2060 (THNHM-I-02392), deposited in THNHM (examined).

##### Non-type material examined.

Malaysia • 1 worker, western Malaysia, Selangor, Ulu Gombak, 22.III.2013, F. Ito leg. (THNHM-I-02465) – Thailand • 2 workers, southern Thailand, Trang Province, Na Yong District, Khao Chong Botanical Garden, 7.XI.2014, W. Jaitrong leg., Colony No WJT071114-2 (THNHM-I-02421 to THNHM-I-02422) • 5 workers, same locality and collector, 26.XII.2018, Colony no. WJT261218-1 (THNHM).

##### Measurements and indices

**(n = 4).**TL 6.63–6.96, HL 1.42–1.45, HW 1.06, SL 1.39–1.42, EL 0.20, ML 2.21–2.31, PL 0.73–0.79, PH 0.53, DPW 0.40, CI 72–74, EI 18, SI 131–134.

##### Description of workers

**(*holotype* and *paratypes*). *Head*** in full-face view subrectangular, clearly longer than broad, sides weakly convex, posterior margin almost straight, posterior corners narrowly rounded. Antenna relatively long, scapes extending beyond posterior corners of head by about 1/4 of their length. Clypeus roughly triangular, median portion distinctly convex, anterior margin broadly convex. Mandibles triangular, masticatory margin with a large apical tooth, followed by 9 or 10 smaller teeth, large and small teeth alternating, basal margin without denticle. Eye slightly convex, located anterior to mid-length of head, relatively large, with 11 ommatidia on the longest axis. Frontal lobes relatively close to each other, with roundly convex lateral margins. Space between frontal carinae distinctly narrowed posteriad.

**Figure 4.**
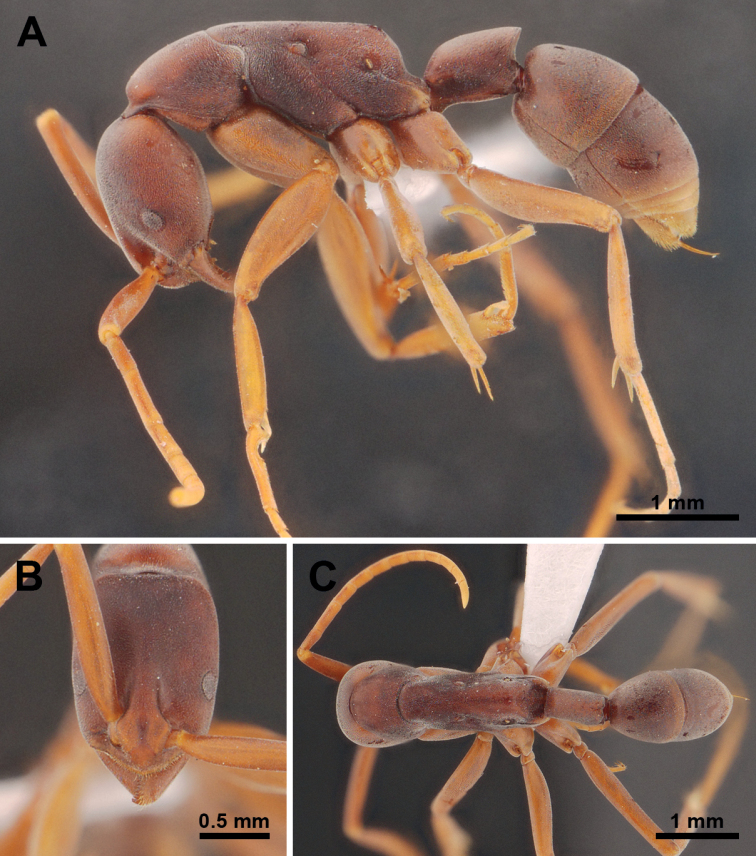
*Platythyreajanyai* (holotype worker, THNHM-I-02392). **A** body in profile view **B** head in full-face view **C** body in dorsal view.

***Mesosoma*** elongate, in profile view pronotum weakly convex, promesonotal suture distinct, dorsal outline of mesonotum and propodeum almost straight, metanotal groove absent, propodeal junction narrowly rounded, declivity weakly convex in its upper half and obviously concave in the lower portion, propodeal spiracle opening elliptical. Legs very long.

***Petiole*** cylindrical and sessile, in profile view clearly longer than high, dorsal outline almost straight, anterodorsal corner broadly rounded, posterodorsal corner extending into an acute angle, posterior margin oblique and weakly concave, ventral margin almost straight with narrowly protruding anteroventral corner; in dorsal view node of petiole rectangular, longer than broad, slightly widening posteriorly, sides almost straight, posterior margin weakly convex with a concavity in the middle.

**Figure 5.**
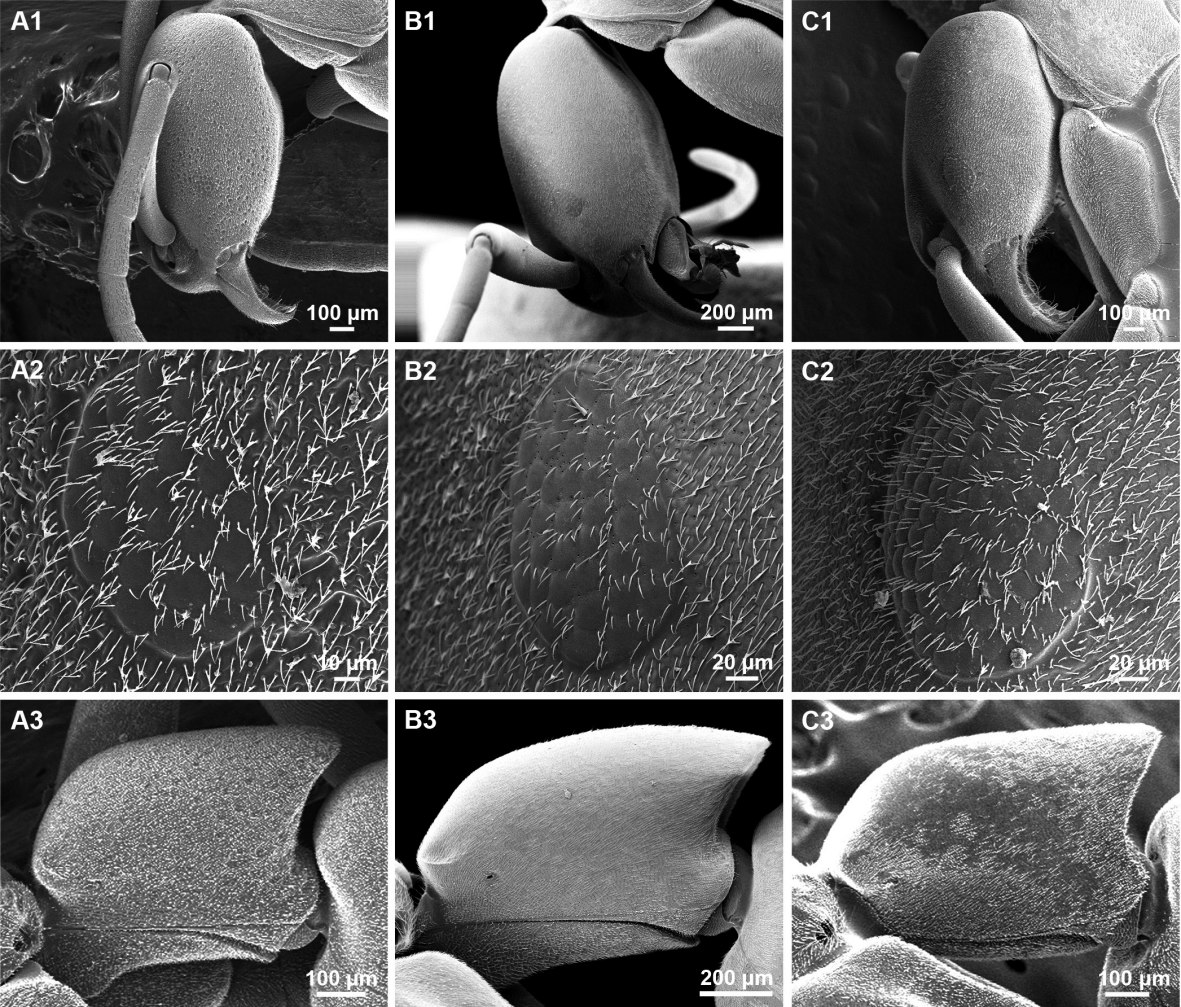
SEM images of *Platythyreaclypeata* (**A1–A3**) *P.homasawini* sp. nov. (**B1–B3**) and *P.janyai* (**C1–C3**). **A1, B1, C1** sculpture on lateral face of head **A2, B2, C2** ommatidia of eye **A3, B3, C3** petiole in profile view.

***Sculpture*.** Head, antennal scapes, mesosoma, petiole and gaster finely densely micropunctate.

***Pilosity*.** The whole body surface and appendages covered with short thick pubescence. Standing hairs absent.

***Coloration*.** Body color reddish brown; antennae, legs, and tip of gaster yellowish brown. Eyes grey. Pubescence white.

##### Distribution.

Thailand (Phatthalung and Trang provinces) and Malaysia (Ulu Gombak National Park) (Fig. 6).

**Figure 6.** Distribution map of *Platythyreaclypeata* species group based on specimens in this study.

##### Comparative notes.

*Platythyreajanyai* is similar to *P.clypeata*, *P.homasawini* and *P.gracillima*. However, *P.janyai* can be easily separated from *P.gracillima* by the following characteristics (characters of *P.gracillima* in parentheses unless otherwise stated): 1) body size smaller (TL = 6.63 mm in *P.janyai*; 9 mm in *P.gracillima*); 2) eye relatively smaller (EL = 0.2 mm in *P.janyai*; 0.3 mm in *P.gracillima*); 3) seen from back, propodeal declivity tapering above (propodeal declivity weakly concave above); 4) petiole laterally convex, seen from above longer than broad (petiole laterally compressed, seen from above a little more than twice as long as broad). For differentiation of *P.janyai* and *P.clypeata*, see “Comparative notes” under *P.clypeata*, and of *P.janyai* and *P.homasawini*, see under *P.homasawini*.

**Figure 7.**
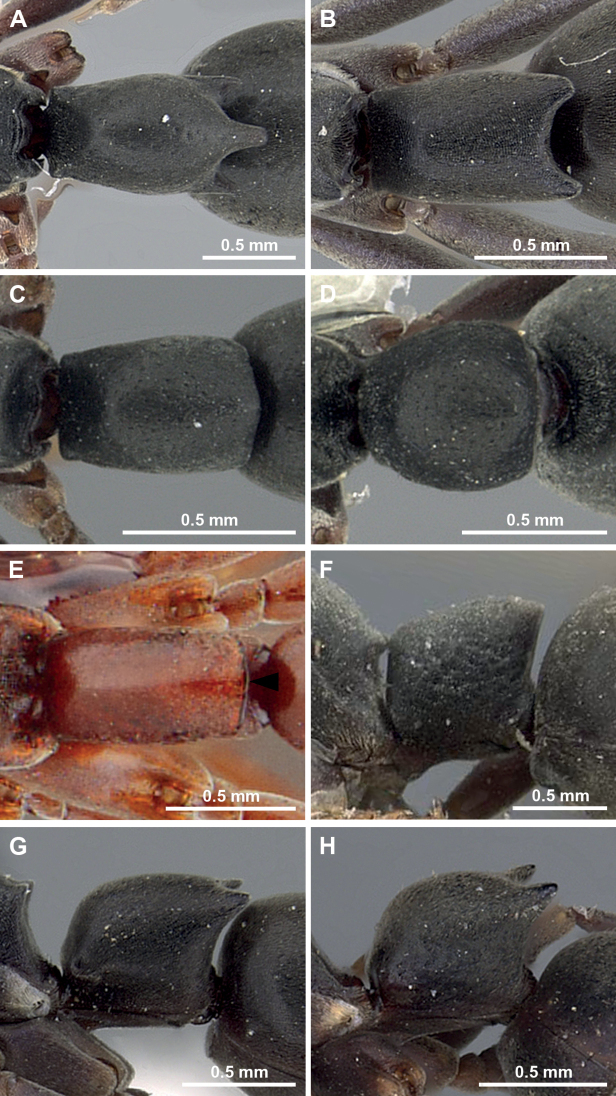
Petiole in dorsal view (**A–E**) and in profile view of *Platythyrea* (**F–H**). **A***P.tricuspidata* (CASENT0281865) **B***P.bidentata* (CASENT0281867) **C***P.parallela* (CASENT0260477) **D***P.inermis* (CASENT0260498) **E***P.homasawini* sp. nov. **F***P.sagei* (CASENT0907117) **G***P.bidentata* (CASENT0281867) **H***P.quadridenta* (CASENT0900569).

**Figure 8.**
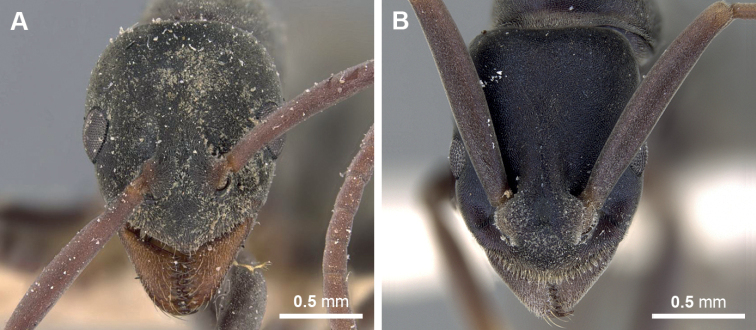
Head in full-face view of *Platythyrea*. **A***P.sagei* (CASENT0907117) **B***P.bidentata* (CASENT0281867).

### ﻿Key to known Oriental species of genus *Platythyrea* based on the worker caste (modified from Phengsi et al. 2018)

**Table:** 

1	Frontal carinae very widely spaced, not continuing beyond level of posterior margin of antennal insertions (Fig. 1A); propodeal spiracle opening circular	**2**
–	Frontal carinae relatively narrowly separated, extending far beyond level of posterior margin of antennal insertions where space between them is very narrow (Fig. 1B); propodeal spiracle opening elliptical	**8**
2	In dorsal view, posterior margin of petiole with 2 or 3 distinct spines, teeth or blunt angles (Fig. 7A, B	**3**
–	In dorsal view, posterior margin of petiole without distinct spines, teeth or sharp angles (Fig. 7C)	**6**
3	In dorsal view, posterior margin of petiole clearly concave with distinct lateral blunt angles; in profile view, petiole almost as long as high	**4**
–	In dorsal view, posterior margin of petiole with 3 distinct spines (Fig. 7A); in profile view, petiole longer than high	***P.tricuspidata* Emery, 1900**
4	In full-face view, head oval, almost as long as broad, posterior margin broadly convex (Fig. 8A); in profile view, posterodorsal corner of petiole nearly right angle (Fig. 7F)	***P.sagei* Forel, 1900**
–	In full-face view, head subrectangular, slightly longer than broad, posterior margin broadly almost straight or weakly concave (Fig. 8B); in profile view, posterodorsal corner of petiole bearing truncate spine (Fig. 7G)	**5**
5	In profile view, lateral face of pronotum punctate, with dense foveae; lateral face of petiole wrinkled; in profile view, dorsal outline of petiole roundly convex (Fig. 7H)	***P.quadridenta* Donisthorpe, 1941**
–	In profile view, lateral faces of pronotum and petiole rather smooth, without foveae; in profile view, dorsal outline of petiole almost straight or weakly convex (Fig. 7G)	***P.bidentata* Brown, 1975**
6	In full-face view, head elongate (CI < 77), posterior margin deeply concave	***P* . *nicobarensis* Forel, 1905**
–	In full-face view, head elongate (CI > 77), posterior margin shallowly concave	**7**
7	In dorsal view, petiole clearly longer than broad (Fig. 7C); antennal scape relatively short, not reaching posterior corner of head	***P.parallela* (Smith, 1859)**
–	In dorsal view, petiole almost as long as broad (Fig. 7D); antennal scape relatively long, slightly extending beyond posterior corner of head	***P.inermis* Forel, 1910**
8	Lateral face of head and pronotum punctate, with dense foveae (Fig. 5A1); antennal scape relatively short (SI = 125–138); smaller species (TL 5.74–6.20; HW 0.86–0.89); eye with 5 or 6 ommatidia on longest axis	***P.clypeata* Forel, 1911**
–	Lateral face of head and pronotum finely micropunctate (Figs 5B1, 5C1); antennal scape relatively long (SI > 150); larger species (TL 7.59–7.80; HW 1.06–1.12)	**9**
9	Head narrower posteriorly (Fig. 3B). Dorsal outline of petiole weakly convex, posterior margin of petiolar node without a concavity in the middle (Fig. 7E). Body surface with thin pubescence (Fig. 3A). Eyes with 7 or 8 ommatidia on longest axis (Fig. 5B2).	***P.homasawini* sp. nov.**
–	Head not narrower posteriorly (Fig. 4B). Dorsal outline of petiole almost straight, posterior margin of petiolar node with a concavity in the middle. Body surface with thick pubescence (Fig. 4A). Eyes with 9–11 ommatidia on longest axis (Fig. 5C2)	***P.janyai* Phengsi et al., 2018**

## ﻿Discussion

With this study, 40 valid species of the genus *Platythyrea* are now known around the world. Among them, six species are found in Thailand, and they belong to two species groups (sensu Brown, 1975): *P.clypeata* group (*P.clypeata*, *P.homasawini* sp. nov., and *P.janyai*) and *P.parallela* group (*P.parallela*, *P.quadridenta*, and *P.tricuspidata*).

The shape of the frontal lobe, mandibular shape and dentition, and shape of mesosoma and petiolar node were characters used by Brown (1975) to distinguish the two species groups mentioned here. These morphological characters were confirmed and used by several authors who described new species after Brown (1975) (Kugler 1977; Lattke 2003; De Andrade 2004; Aria et al. 2011; Phengsi et al. 2018). The present study follows the previous works and also proposed the peculiar shape of the propodeal spiracle, frontal carina condition, and shape of propodeal junction as more important characters to separate the two species groups.

The *P.clypeata* group can be distinguished from other congeners mainly by the following: body reddish brown; space between frontal carinae distinctly narrowed posteriad; propodeal spiracle opening elliptical, petiole longer than high, and posterior margin concave. The worker caste of *Platythyrea* species is generally monomorphic with little variation in size within species. Thus, worker body size can be used for separating large and small species. In this study, worker body size has been used to separate *P.homasawini* sp. nov., *P.janyai*, and *P.clypeata*.

*Platythyreaclypeata* is distinctly allopatric with *P.janyai* in geographic subregion in continental Asia (Phengsi et al. 2018). It has been recorded from Sri Lanka, Vietnam, Laos, and eastern Thailand (Brown 1975), whereas *P.janyai* occurs on the Malay Peninsula (South Thailand and West Malaysia). However, *P.clypeata* and *P.janyai* both were found in lowland (< 300 m a.s.l.). *Platythyreaclypeata* inhabits various habitats including plantations, secondary forests, and primary dry evergreen forests. *Platythyreajanyai* is confined to primary evergreen forests. *Platythyreahomasawini* sp. nov. is restricted to highland hill evergreen forests.

## Supplementary Material

XML Treatment for Platythyrea clypeata

XML Treatment for Platythyrea homasawini

XML Treatment for Platythyrea janyai
